# The Effect of Viscous Drag on the Maximum Residual Stresses Achievable in High-Yield-Strength Materials in Laser Shock Processing

**DOI:** 10.3390/ma16216858

**Published:** 2023-10-25

**Authors:** Ignacio Angulo, Wsewolod Warzanskyj, Francisco Cordovilla, Marcos Díaz, Juan Antonio Porro, Ángel García-Beltrán, José Luis Ocaña

**Affiliations:** UPM Laser Centre, Polytechnical University of Madrid, C/Alan Turing 1, 28031 Madrid, Spain; wsewolod.warzanskyj@upm.es (W.W.); francisco.cordovilla.baro@upm.es (F.C.); marcos.diaz@upm.es (M.D.); japorro@etsii.upm.es (J.A.P.); agarcia@etsii.upm.es (Á.G.-B.); jlocana@etsii.upm.es (J.L.O.)

**Keywords:** shock loading, laser shock processing, high strain rates, plastic deformation model, residual stresses

## Abstract

In this paper, the experimentally observed significant increase in yield stress for strain rates beyond 10^4^ s^−1^ (viscous regime) is explicitly considered in laser shock processing (LSP) simulations. First, a detailed review of the most common high-strain-rate deformation models is presented, highlighting the expected strain rates in materials subject to LSP for a wide range of treatment conditions. Second, the abrupt yield stress increase presented beyond 10^4^ s^−1^ is explicitly considered in the material model of a titanium alloy subject to LSP. A combined numerical–analytical approach is used to predict the time evolution of the plastic strain. Finally, extended areas are irradiated covering a squared area of 25 × 25 mm^2^ for numerical–experimental validation. The in-depth experimental residual stress profiles are obtained by means of the hole drilling method. Near-surface-temperature gradients are explicitly considered in simulations. In summary, the conventionally accepted strain rate range in LSP (10^6^–10^7^ s^−1^) is challenged in this paper. Results show that the conventional high-strain-rate hardening models widely used in LSP simulations (i.e., Johnson Cook model) clearly overestimate the induced compressive residual stresses. Additionally, pressure decay, whose importance is usually neglected, has been found to play a significant role in the total plastic strain achieved by LSP treatments.

## 1. Introduction

Nowadays, there is an increasing interest in improving the properties of metal material alloys. Typically, many manufacturing processes, including some modern ones such as additive manufacturing (AM), implicitly involve the appearance of surface tensile residual stresses. Mechanical processing (bending and rolling), phase transformations and strong thermal variations (for instance, derived from AM and welding) frequently lead to tensile residual stresses and changes in the material properties, as documented in [1,2,3,4]. These tensile stresses imply a notable reduction in the fatigue life and, consequently, there is a growing interest in research into surface treatments that deal with this issue. LSP is a mechanical surface treatment in which the material is deformed by the effect of high-intensity shockwaves propagating through the material. In LSP, the surface of the material is irradiated with a high intensity (GW/cm^2^) pulsed laser beam with a full width at half maximum (FWHM) lower than 50 ns [5,6]. The high-intensity irradiation forces a sudden vaporization of a thin layer, developing an ionized plasma at high pressures with the aid of a confining medium (typically water or quartz glass). Typically, the magnitude of the shockwave generated (about 5 GPa) is capable of deforming metallic alloys from the surface up to 1 mm depth [7], introducing in-depth compressive residual stresses. This generates a protective layer which finally develops a fatigue life improvement [8,9,10].

Due to the proven ability of LSP to enhance fatigue life [8,11,12,13,14,15,16], there is intensive research focused on deepening the knowledge of the physics involved in LSP. This way, the costly experimental trial and error to achieve a successful fatigue life enhancement can be reduced drastically. The experimental residual stress measurement methods are of great importance in this area. These methods can be classified as destructive (contour method) [17], semi-destructive (hole drilling method) [18] and non-destructive (X-ray diffraction, neutron diffraction, and ultrasonic testing) [19]. By means of the contour method, a complete residual stress map can be obtained inside the sectioned area. However, experimental evidence shows softened in-depth stress profiles when high stress gradients are presented. With the aid of the hole drilling method, the general trendline in the in-depth residual stress profile up to 1 mm can be obtained. However, this method is quite sensitive to surface detection, leading to high uncertainties near the surface. The X-ray diffraction method provides relatively precise near-surface stress measurements, which completes the results obtained by the hole drilling method.

Given the multiphysics nature of LSP treatment, its predictive characterization is complex. The main physical phenomena range from laser–plasma interaction to shockwave propagation through the material, and hence multiple parameters need to be modeled. Furthermore, the numerical–experimental validation of each phenomenon is quite difficult and frequently material models are calibrated just to ensure good in-depth residual stress predictions. Consequently, the proper description of the physical basis involved, which is essential for scientific progress, is often neglected. Usually, the mechanical response in specimens subject to LSP has been modeled considering relatively low sensitivity to the strain rate (i.e., the Johnson–Cook model). This implies that both analytical models and numerical simulations predict deformation rates in the order of 10^6^–10^7^ s^−1^, which is inconsistent in general terms with the fact that an abrupt yield stress increase is experimentally observed beyond 10^4^ s^−1^ [20]. This is the main point discussed in this paper.

Concerning the development of strain-rate-dependent models, a large number of research lines are aimed at studying the effect of the strain rate in a wide range of conditions, from quasistatic (10^−3^ s^−1^) to ultrahigh strain rates (10^8^ s^−1^). The constitutive strain-rate-dependent models developed in the last years can be essentially divided into two groups: phenomenological models (the Johnson–Cook model [21], Khan–Huang–Liang (KHL) models [22,23,24,25] and a recent model presented by Kim [26]) and physically based models, for FCC (face-centered cubic), BC (centered cubic) and HCP (hexagonal close-packed) structures [27,28,29,30]. Phenomenological models are focused on achieving the best fit option between numerical predictions and experimental stress–strain curves, with no strict physical interpretation of the calibration constants. The material behavior is then modeled with a minimization of the required constants for calibration. On the other hand, the physically based models are focused on the strict physical interpretation of each calibration constant. However, a great number of constants are usually involved. In addition, the proper identification of them is often difficult to assess. Hence, very different sets of constants are reported in the literature for the same material, leading to inconsistent results. The recently proposed physical-based models consider explicitly the abrupt yield stress increase at 10^4^ s^−1^. This noticeable yield stress has been documented by Couque [20], providing a precise description of the methods to characterize the strain rate behavior beyond 10^3^ s^−1^ with the direct impact Hopkinson pressure bar (DIHPB) technique [31] in copper, nickel, AISI 304L steel, Al 2017 aluminum alloy, tantalum and two tungsten alloys. Additionally, the strain rate threshold between the thermal activation regime and the viscous regime is identified for each material. The conventional Johnson–Cook model considers the yield stress as proportional to a strain rate term. It models the linear strain rate dependence experimentally observed from quasistatic conditions (${\dot{\varepsilon }}_{p}≅$ 10^−4^ s^−1^) up to moderated dynamic conditions (${\dot{\varepsilon }}_{p}≅$ 10^3^ s^−1^). However, beyond dynamic conditions, plasticity is governed by a viscous drag mechanism: the dislocation motion is slowed down by a viscous drag phenomenon. Hence, dislocation motion, with the required conditions for its thermal activation, becomes a minor effect in plasticity. This leads to a drastic increase in the yield stress at very high-strain-rate conditions (${\dot{\varepsilon }}_{p}$ > 10^4^ s^−1^) which can be hardly modeled by the Johnson–Cook formulation.

New models have been proposed to deal with this issue, for instance, a modified version of the Johnson–Cook model [32], Zerilli–Armstrong model [30], and Gao–Zhang models [27,28,29]. Most of the Zerilli–Armstrong model parameters can be correlated with physical material constants. This represents a substantial advantage. In addition, the material’s crystalline structure (BCC, FCC and HCP) is explicitly considered. Plasticity in BCC structures is governed by Peierls–Nabarro barriers [33]. Thus, the stress associated with thermal activation is independent of the accumulated plastic strain. Plasticity in FCC materials is determined by forest dislocation motion [34] and thermal activation is linked to plastic strain. In HCP crystalline structures, both BCC and FCC deformation mechanisms coexist. Hence, Zerilli–Armstrong proposes a linear combination of BCC and FCC formulations, leading to a complex constitutive model that includes 15 calibration constants to be determined. Therefore, model calibration is difficult to approach, leading to high-computational-cost simulations. Gao–Zhang presented a comprehensive analysis of the physical phenomena which take place from quasistatic up to high strain rates for HCP structures [27] and FCC structures [28]. Then, a latter formulation was developed to model the viscous regime [29]. The yield stress is then composed of three main components, including a drag stress term, $\sigma_{drag}=a{\left({\dot{\varepsilon }}_{p}\right)}^{n_{2}}$. All the described models are conceived to deal with the abrupt yield stress increase presented near 10^4^ s^−1^. For higher strain rates in shock loading experiments, Swegle and Grady [35] proposed the exponential form $\sigma ≅K{\dot{\varepsilon }}_{p}^{q}$ $\left(q=1/4\right)$, to achieve a best fit option for six different metals in the range 10^5^–10^8^ s^−1^. An additional trend change is documented beyond 10^8^ s^−1^, which can be properly simulated with dislocation dynamics [36]. The exponential factor is then recalibrated $\left(q=1/2\right)$.

In the predictive characterization of LSP, the use of a unique linear strain-rate-dependent function (i.e., the Johnson–Cook model) is still widely accepted. This function is usually calibrated up to dynamic conditions (10^3^ s^−1^). The trend of the curve is extrapolated for higher strain rates. This leads to predicted strain rates in LSP which are about 10^6^ s^−1^, as documented in the literature [37,38,39,40]. However, this issue is challenged by solid theoretical arguments. Simon et al. [41] reported the limitations of the Johnson–Cook model to provide accurate stress–strain predictions over a wide range of temperatures and strain rates. The experimental results presented in their publication showed a significant increase in the stress rate sensitivity beyond 10^3^ s^−1^ for a high-strength steel. Furthermore, a recent publication explicitly considered the abrupt yield stress increase in LSP-treated OFHC (oxygen-free high thermal conductivity) copper specimens [42], which shows a good numerical–experimental agreement in the deformed profiles for the application of concentric shots. The in-depth Vickers hardness is also estimated with good precision. A later publication provided a numerical study of LSP in Ti6Al4V alloy, in which a dislocation density evolution model [43] was calibrated on the basis of the results provided by the Gao–Zhang model [27]. However, the original Gao–Zhang model [27] was updated in 2019 with the addition of a drag stress term since the abrupt yield stress increase was underestimated [29]. Consequently, a detailed description of the time evolution of the plastic strains and an experimental validation of the in-depth residual stresses are still required. This is especially relevant for the study of very high-density LSP treatments.

The results reported in the literature show relatively precise FEM (finite element method) estimations by means of the Johnson–Cook model in LSP. However, the nature of the deformation mechanisms involved is not explicitly considered. In fact, several authors proposed a recalibration in the Johnson–Cook model parameters in LSP-treated Ti6Al4V specimens by means of an inverse approach [44]. This led to a significant increase in the recalibrated linear strain rate sensitivity (calibrated originally up to 10^3^ s^−1^, by means of the split-Hopkinson bar (SHB)). This significant difference was precisely attributed to the abrupt yield stress increase presented at 10^4^ s^−1^. This last result may be interpreted as evidence of the existence of this phenomenon in specimens subject to LSP.

The present study starts with a theoretical background analysis providing solid arguments that justify the relevance of considering the viscous drag phenomenon in LSP (Section 2). Hence, the developed methods for its precise simulation and experimental validation are described (Section 3). Significant differences are presented between LSP-treated materials with low and high yield stresses. Finally, the nature of the computed plastic strains is studied with the application of concentric pressure pulses (Section 4.1) and in realistic high-density LSP treatments, in which extended areas are irradiated for numerical–experimental validation (Section 4.2).

In summary, the conventionally accepted strain rate range in LSP (10^6^–10^7^ s^−1^) is challenged in this paper. The results show that the conventional high-strain-rate hardening models widely used in LSP simulations (i.e., the Johnson–Cook model) clearly overestimate the induced compressive residual stresses, whereas the natural residual stress saturation widely reported in experiments can be properly modeled if viscous drag is considered. Furthermore, pressure decay, whose importance is usually neglected, has been found to play a significant role in the total plastic strain achieved by LSP treatments. Overall, it is expected that the present advances derived from the explicit consideration of viscous drag will represent a starting point of interest for future research.

## 2. Theoretical Basis: On the Consideration of the Viscous Drag Mechanism in Metal Material Alloys Subject to LSP

Although the viscous drag mechanism has been documented in previous studies, an exhaustive analysis of its impact on deformation mechanisms is currently required in LSP modeling. This section starts by introducing a generalization of the conventional analytical–numerical methods to consider the abrupt yield stress increase in LSP (Section 2.1). Then, a general description of the expected response of low/high-yield-stress alloys subject to LSP is presented (Section 2.2).

### 2.1. Generalization of the Conventional Analytical Methods to Consider the Abrupt Yield Stress Increase in Materials Subject to LSP

In LSP, the material is subject to high-amplitude shockwaves, experiencing a phenomenon called uniaxial strain. The results presented in [7,45] show that the computed axial strain is proportional to the difference between the peak pressure, $P_{max}$, and the Hugoniot elastic limit, $\sigma_{H}$. The Johnson–Cook model computes low variations in the yield stress from quasistatic conditions to ultrahigh strain rates usually reported (${\dot{\varepsilon }}_{p}≅$ 10^6^–10^7^ s^−1^). Therefore, the Hugoniot elastic limit, $\sigma_{H}$, remains practically constant during the deformation process. A generalized form of the original equation is proposed to compute the temporal significant variation in the Hugoniot elastic limit (Equation (1)): a functional form, $\sigma_{H}=f({\dot{\varepsilon }}_{pz})$, is considered in material modeling, where ${\dot{\varepsilon }}_{pz}$ represents the axial plastic strain rate. Hence, the time evolution of the strain rate can be calculated. For relatively low strain rates, the axial plastic strain can be approximated using an integral expression (Equation (2)). If the viscous drag phenomenon is neglected, the Hugoniot elastic limit remains practically constant (${\dot{\sigma }}_{H}≅0$), and then the integration of Equation (1) leads to the conventional analytical expression widely used for plastic strain determination. The maximum plastic strain rate computed (${\dot{\varepsilon }}_{p}≅$ 10^6^–10^7^ s^−1^) may be presented for alloys characterized by relatively low yield stress during the first nanoseconds, where $\dot{P}\left(t\right)$ reaches its maximum value (about 1 GPa/ns) and ${\dot{\sigma }}_{H}$ progressively decreases down to zero.

(1) $${\dot{\varepsilon }}_{pz}\left(t\right)=-\frac{2\left(1-2\upsilon \right)}{E}\left(\dot{P}\left(t\right)-{\dot{\sigma }}_{H}\right)$$

(2) $$\varepsilon_{pz}=-\int_{0}^{t_{end}}f^{-1}\left(\sigma_{H}\right)dt≅-\int_{0}^{t_{end}}f^{-1}\left(P\left(t\right)\right)dt$$

where: $\varepsilon_{pz}\equiv$ axial plastic strain; $P\left(t\right)\equiv$ time pressure evolution; $\sigma_{H}\equiv$ Hugoniot elastic limit; E≡ Young’s modulus; υ≡ Poisson’s ratio; $t_{end}\equiv$ time at which pressure is extinguished.

### 2.2. Expected Response in Low/High-Yield-Stress Alloys Subject to LSP

The nature of LSP needs to be explicitly considered in material modeling to account properly for the viscous drag mechanism. Significant differences may be presented between low- and high-yield-stress alloys. In general terms, the magnitude of the maximum achievable peak shockwave amplitude (about 5–6 GPa) may be enough to reach the conventionally reported plastic strain rates (about 10^6^ s^−1^) for relatively low-yield-stress alloys (for instance, copper), in a consistent way with experimental results obtained using the SHB. In fact, a best fit in the range 10^6^–10^7^ s^−1^ with Swegle and Grady’s exponential form $\sigma ≅K{\dot{\varepsilon }}_{p}^{q}$ proposed in [46] (where q = 1/3 for aluminum and 1/2.32 for copper) shows that the Hugoniot elastic limit expected at 10^6^ s^−1^ is below the peak pressure, and, consequently, the strain rates in LSP may reach 10^6^ s^−1^. On the other hand, the combination of a high yield stress (typical in titanium alloys) and the abrupt yield stress increase at the viscous regime (about 10^4^ s^−1^) imposes a limited maximum achievable plastic strain rate (about 10^4^ s^−1^) for the maximum shockwave pressures (about 5–6 GPa) generated by LSP. This limited plastic strain rate leads to a considerable reduction in the conventionally expected computed plastic strains. Nevertheless, this issue does not imply any inconsistency with the experimental results: very small plastic strains are required for generating notable in-depth residual stress profiles. In fact, plastic strains below 1% are enough to achieve significant compressive residual stresses according to the generalized Hooke’s law. Negligible hardening is expected for conventional low-density treatments and hence it may be suitable to adopt an elastic–perfectly plastic model for simulations.

## 3. Materials and Methods

This section provides both a detailed description of the experimental set-up for validation (Section 3.1) and the corresponding FEM model definition (Section 3.2). Ti6Al4V is selected for the present study as a representative high-yield-stress alloy. High-density treatments are developed in a centered squared area of 25 × 25 mm^2^. This surface is enough to measure thein-depth residual stress profiles by means of the hole drilling method.

### 3.1. Experimental Set-Up for LSP Irradiation and Measurement of In-Depth Residual Stresses

The Ti6Al4V samples are provided in rolled plates of 50 × 50 × 7 mm^3^ (Table 1). The specimen is thick enough (≅7 mm) to prevent it from severe residual stress redistribution due to specimen bending. All the specimens were subjected previously to a thermal relaxation cycle (710 °C for 2 h) to suppress any tensile residual stress derived from the manufacturing process. Additionally, this leads to a reduction in the stress–strain asymmetry.

**Figure 1:**
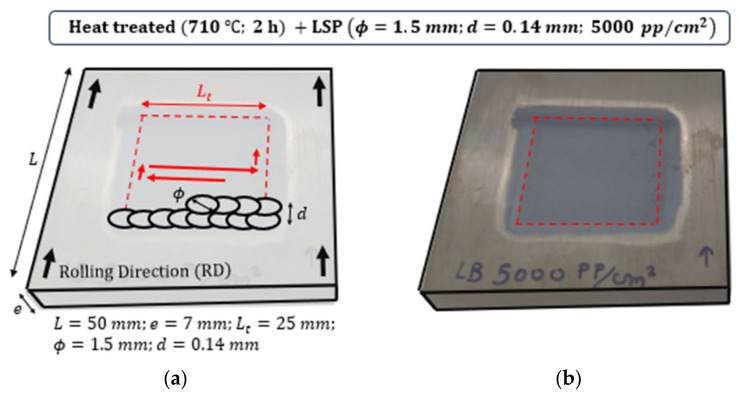
(**a**) Schematic representation of the treatment strategy. (**b**) Experimental result after irradiation.

The laser device in the present study is a Q-switched Nd:YAG that provides 2.4 J per pulse and operates at 10 Hz with a FWHM of 9 ns. A thin layer of water has been used as the confining media. The laser spot diameter is set to ϕ=1.5 mm, leading to a peak laser intensity of I = 20 GW/cm^2^ and a peak pressure of 5.3 GPa, calculated with the aid of HELIOS code and the methods presented in [47]. The overlapping distance is set to d=0.14 mm, leading to an equivalent overlapping density [48] (EOD)= 5000 pp/cm^2^. A schematic representation of the treatment strategy and a picture of the result after irradiation is represented in Figure 1. The laser spot diameter, ϕ, and overlapping distance between successive pulses, d, are not represented at scale to provide a clearer understanding of the treatment strategy.

The experimental in-depth residual stress profiles for validation have been obtained by means of the hole drilling method (ASTM E837-13a) [18]. It consists of drilling the material progressively up to 1 mm depth. Then, a specific strain rosette registers the local deformations nearby the hole, caused by the removal of stressed material. Finally, the residual stresses are obtained by means of a specific algorithm. The optimal depths for calculation have been selected according to reference [49]. Four measurements have been taken inside the irradiated area. The in-depth residual stress is then obtained by averaging.

### 3.2. FEM Model

#### 3.2.1. Model Definition for LSP Simulation

LSP is modeled by means of a spatial–temporal pressure pulse profile, $P\left(r,t\right)$, in combination with a heat flux exchange between the confining plasma and the surface of the material. The spatial–temporal pressure pulse profile, $P\left(r,t\right)$ is defined by Equation (3). Both the time pressure function, $P\left(t\right)$, and the quasi-Gaussian spatial distribution has been extracted from reference [47], where $a_{\phi }$ = 1.81, G = 3.2, R = 0.75 mm and r represents the distance between a generic point to the center of the laser spot.

(3) $$P\left(r,t\right)=P\left(t\right)e^{-a_{\phi }{\left(\frac{r}{R}\right)}^{G}}$$

**Figure 2:**
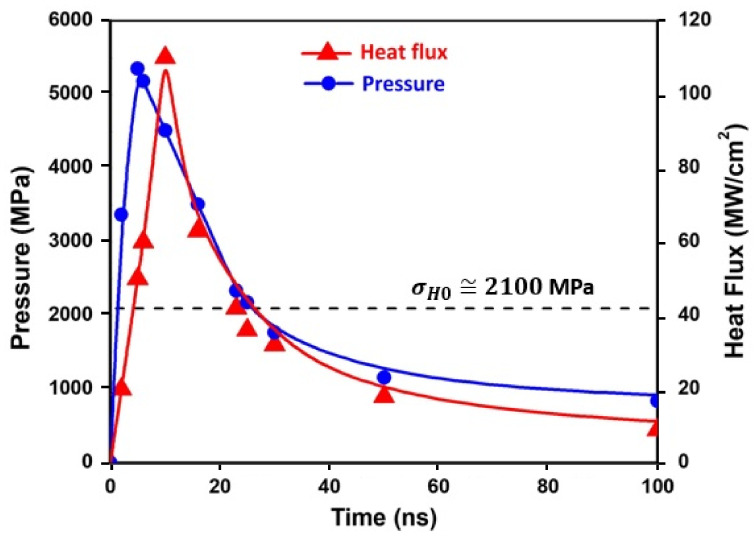
Time evolution of the heat flux and pressure.

The application of a peak laser intensity of 20 GW/cm^2^ forces a sudden vaporization of the irradiated surface, leading to a very high-temperature plasma (about 30,000 K). This causes additional local near-surface plastic strains. The time–heat flux exchange between the confined plasma and the irradiated surface can be calculated with the aid of the HELIOS code. The time irradiation pulse, $I\left(t\right)$, the heat flux, $q\left(t\right)/A$, and the corresponding pressure, $P\left(t\right)$, are represented in Table 2. Both the heat flux, $q\left(t\right)/A$, and the pressure, $P\left(t\right)$, are plotted together in Figure 2, in which a slight delay is observed between both profiles. This is consistent with the results reported by Morales et al. [50]. The pressure pulse is set in ABAQUS with the aid of user subroutine VDLOAD.

The simulations have been performed using a 3D FEM model by means of commercial software (ABAQUS (version number: 6.14)). The material model definition can be set either with an explicit definition (an equation defined in a user subroutine) or directly defining the yield stress dependence in a table in a simpler way. Hence, the computed yield stress by means of the Gao–Zhang model has been set for discrete increments of plastic strain, $\varepsilon_{p}$, plastic strain rate, ${\dot{\varepsilon }}_{p}$, and temperature, T, to cover the whole range of expected conditions during shock loading (Equation (4)). These discrete increments for table generation have been carefully selected to guarantee a convergence in results.

(4) $$\sigma_{y}=f\left(\varepsilon_{p},\;{\dot{\varepsilon }}_{p},T\right)=f\left(n_{1}∆\varepsilon_{p},\;n_{2}∆{\dot{\varepsilon }}_{p},T_{0}+n_{3}∆T\right)\;\;$$

where: $n_{1}=0,1\ldots 8;$ $∆\varepsilon_{p}=0.025$; $n_{2}=0,1\ldots 20;$ $∆{\dot{\varepsilon }}_{p}=100\;s^{-1}$; $n_{3}=0,1\ldots 6;$ ∆T=100 K; $T_{0}=300\;K$.

The mesh consists of 100 × 100 × 10 μm^3^ hexahedral elements with a reduced integration scheme (C3D8R), in which the minimum dimension corresponds to the in-depth dimension (10 μm). The mesh is surrounded by infinite elements (CIN3D8) covering a total depth of 7 mm. The minimum step time provided by the FEM code that ensures stable solutions in the explicit algorithm is $t_{s}≅$ 1.6 ns. This is the result of the application of the Courant–Friedrichs–Lewy (CFL) condition and a safety factor which is set automatically by the FEM code. However, $t_{s}$ has been reduced to 0.1 ns. This way, a precise characterization of the deformations in the range 10^−4^ to 10^4^ s^−1^ is computed, although the computational cost rises. The laser device operates at 10 Hz, leading to a step time of $t_{p}=$ 0.1 s between consecutive pulses. Nevertheless, postprocessing results analysis shows that the stresses are balanced beyond 2 μs. Hence, the time between consecutive pulses is set to $t_{p}=2\;\mu s$ to reduce the computational cost. 

To reach an efficient solution, a reduced representative irradiated patch of 3 × 3 mm^2^ is finally used for the simulations, which is enough to ensure a sufficient overlapped area. Results may differ slightly from a complete 25 × 25 mm^2^ treatment simulation. Nevertheless, minor differences are expected considering the small magnitude of predicted plastic strains. Considering both the treatment density (5000 pp/cm^2^) and the representative patch area (3 × 3 mm^2^), a total amount of $n_{p}=$ 450 shots need to be simulated. Consequently, the simulation time responds to $t_{sim}=$ $n_{p}t_{p}=$ 0.0009 s and the number of step increments for simulation is $n_{sim}=9000$.

#### 3.2.2. Near-Surface Thermal Effect Simulation (Implicit Analysis)

Concerning the simulation of thermal effects, a finer mesh is used (≅0.8 μm). Only a few microns exhibit high temperatures during pressure decay. This leads to a temporal reduction in the yield stress in combination with drastic local thermal expansions. Consequently, severe local reverse yielding is presented at the surface. Hence, plastic strains and the magnitude of the compressive residual stresses are strongly affected at the first microns. In fact, radial plastic strains at the surface become negative and tensile residual stresses are presented. This is consistent with previously reported thermomechanical studies in LSP treatments [50,51]. Very high temperatures are presented during irradiation (in fact, a small volume of material is evaporated). However, the heat-affected layer is very small, and hence the time between successive irradiations (0.1 s) is enough for the material to reach almost room temperature. Therefore, the complete simulation process can be divided into two steps: a mechanical process (shockwave propagation), followed by a near-surface thermal simulation (with an implicit scheme). A simplified method is used to calculate the material vaporization: if the surface element affected by irradiation reaches the vaporization temperature of the alloy (≅3500 K), the heat flux starts to affect the adjacent element.

The plastic strains obtained using shock loading are extracted and set as an input for thermal simulation with the aid of the eigenstress method: the elastic self-balanced solution is then represented by setting artificially orthotropic thermal expansion coefficients in combination with a fixed temperature as a boundary condition. The time evolution of the temperature has been obtained using a previous heat flux analysis with the aid of the data presented in Table 2. Considering that the temperature has been reserved and used to achieve artificially a realistic balanced solution, a user-defined field variable FV1 plays the role of the realistic temperature evolution. Therefore, the hardening model definition at this step is obtained replacing T with FV1 in Equation (4).

## 4. Results

Concerning all the points discussed above, Ti6Al4V is selected as a suitable candidate for studying the impact of the abrupt yield stress increase in LSP: the experimental strain rate characterization presented in the literature shows that the yield stress of the material reaches about 2 GPa (equivalent to a Hugoniot elastic limit of about 4 GPa) near the strain rate threshold between the thermally activation regime and viscous regime (10^4^ s^−1^). Therefore, a Hugoniot elastic limit for the achievable pressures in LSP may be presented precisely near this strain rate threshold. Consequently, significant differences are expected with respect to the results provided by conventional modeling. Setting a small spot diameter, ϕ=1.5 mm, ensures the highest pressures, which may be necessary considering the high yield stress of Ti6Al4V alloy.

In the first Section 4.1, a detailed numerical study for the application of single shots is presented. Then, a coupled FEM thermomechanical model is applied for the determination of the in-depth residual stress profiles in realistic high-coverage LSP treatments (Section 4.2). Additionally, the effect of the high temperatures involved in near-surface stresses is discussed. The simulated results are validated by means of the experimental in-depth profiles obtained using the hole drilling method.

### 4.1. Analytical–Numerical Results for LSP Single Shots in Ti6Al4V

The surface plastic strains are calculated for the application of a single shot. The main differences when considering the effect of viscous drag are studied both in a qualitative and quantitative way. The conventional Johnson–Cook model is considered with its corresponding conventional calibration parameters presented by Lesuer [52]. The modified Gao–Zhang formulation is selected as representative for drag stress modeling in LSP [29] in the range of 10^−4^ to 10^4^ s^−1^. As discussed previously, the material is not expected to reach the typical reported strain rates in LSP (10^6^ s^−1^) for Ti6Al4V alloy.

The time pressure evolution, $P\left(t\right)$, has been documented in Section 3 (Figure 2 and Table 1). The maximum pressure is $P_{max}≅$ 5.3 GPa at 5 ns. Hence, a maximum achievable plastic strain rate of $\left|{\dot{\varepsilon }}_{pzmax}\right|≅$ 7 · 10^6^ s^−1^ is computed using Equation (1), setting ${\dot{\sigma }}_{H}=0$. This scenario corresponds to the conventionally reported strain rates in LSP reported in the literature.

However, the abrupt yield stress presented at 10^4^ s^−1^ in experimental curves of Ti6Al4V suggests that a Hugoniot elastic limit about the maximum pressure ($\sigma_{H}=P_{max}≅$ 5.3 GPa) may be presented approximately about 2 × 10^4^ s^−1^. Consequently, this imposes a limit on the plastic strain rate, and much higher pressures would be required to achieve $\left|{\dot{\varepsilon }}_{pzmax}\right|$. The computed axial plastic strain by means of the Johnson–Cook model, $\varepsilon_{pz\;free}$, and Gao–Zhang model, $\varepsilon_{pz\;Vd}$, is presented in Equations (5) and (6). As expected, significant differences are computed between both calculations.

(5) $$\varepsilon_{pz\;free}≅-\frac{2\left(1-2\upsilon \right)}{E}\left(P_{max}-\sigma_{H}\left({\dot{\varepsilon }}_{pz}=7\times 10^{6}\;s^{-1}\right)\right)≅-0.016$$

(6) $$\varepsilon_{pz\;Vd}≅-\int_{0}^{t_{end}}f^{-1}\left(P\left(t\right)\right)dt≅-0.00036$$

**Figure 3:**
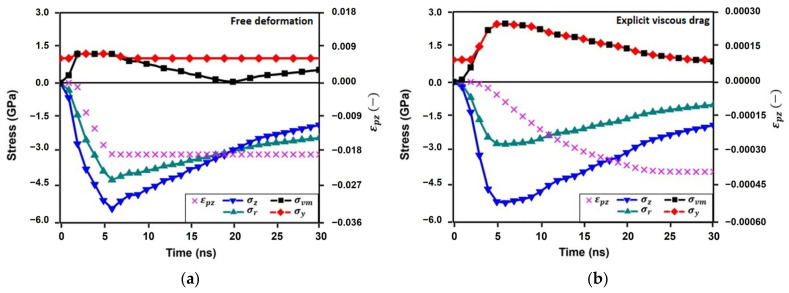
(**a**) FEM results predicted using conventional model. (**b**) FEM results predicted with explicit consideration of viscous drag.

These analytical formulations provide a tentative estimation of the achieved near-surface plastic strains after one single shot. Realistic FEM simulations are required for more accurate predictions. Figure 3 shows the time evolution of the calculated axial stress, $\sigma_{z}\left(t\right)$, the radial stress, $\sigma_{r}\left(t\right)$, the von Mises stress, $\sigma_{vm}\left(t\right)$, the dynamic yield stress, $\sigma_{y}\left(t\right)≅\sigma_{H}\left(t\right)/2$, and the axial plastic strain, $\varepsilon_{pz}\left(t\right)$, computed by the FEM simulations by means of both models. The axial plastic strains calculated using the FEM simulations, $\varepsilon_{pz}\left(t\right)$, confirm the accuracy of the analytical predictions: converged plastic strains $\varepsilon_{pz\;free}=-0.019$ and $\varepsilon_{pz\;Vd}=-0.00040$. Hence, the relative errors between the analytical and numerical simulations are about 15% and 10%, respectively.

The results presented in Figure 3b lead to a significant conclusion: most plastic straining is presented during pressure decay. This pressure decay plays an important role in plastic straining when the abrupt yield stress increase is considered. The material does not reach the maximum achievable plastic strain rate imposed by the pressure raise (about 7 × 10^6^ s^−1^). Consequently, the proper simulation of plasma dynamics is essential to identify both the maximum pressure, and the corresponding nature of the time profile beyond this maximum. The plastic strains achieved are proportional to the difference $P_{max}-\sigma_{H0}$ and the time in which the pressure decay is presented.

On the other hand, most of the plastic straining is computed during pressure increase if viscous drag is neglected. Notable plastic strains are computed (Figure 3a) and then the study of the nature of the pressure decay becomes a minor relevant issue. It is important to note that the results presented in Figure 3a have a double purpose: first, to show the differences when the yield stress is underestimated by means of conventional modeling, and second, to show the representative realistic deformation mechanism for low-strength metals, like copper and aluminum. In a qualitative way, all conclusions and trends deduced using conventional modeling in Ti6Al4V may be then extrapolated to low-strength alloys subject to LSP.

**Figure 4:**
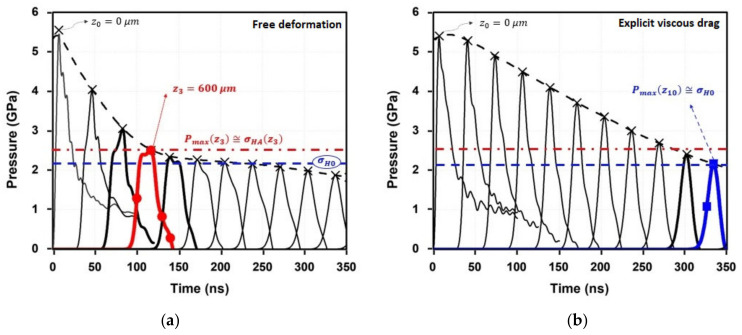
(**a**) Time shockwave evolution for representative depths computed by conventional model. (**b**) Results with explicit consideration of viscous drag.

**Figure 5:**
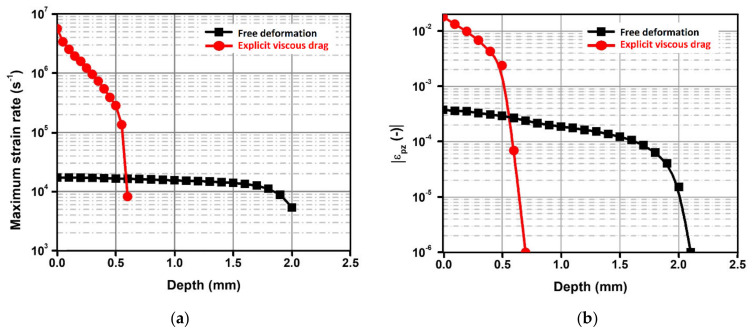
(**a**) In-depth maximum plastic strain rate. (**b**) In-depth axial plastic strain.

Figure 4 shows the computed time evolution of the shockwave amplitude for the selected representative depths. A linear decrease in the maximum pressure, $P_{max}\left(z_{i}\right)$, is computed by means of conventional modeling from the surface of the material, from $z_{0}$ (where $P_{max}\left(z_{0}\right)≅$ 5.3 GPa) to $z_{3}$ = 600 μm (where the strain rate decreases drastically). Furthermore, the results provided when the drag stress is considered (Figure 4b) show that the in-depth pressure decay is softened. As expected, the shockwave loses a greater amount of energy due to the higher magnitude of plastic deformation when the viscous drag is neglected. Therefore, the plastic strain near the surface is higher but a lower affected depth is computed (Figure 5).

In summary, the results presented in this section confirm both qualitative and quantitative differences are presented when the viscous drag is explicitly considered. The pressure decay beyond the maximum shockwave amplitude plays a very important role in plastic deformation. In fact, it is responsible for extending the time in which deformation rates about 10^4^ s^−1^ take place. This fact justifies that reasonable deformations are obtained (with their corresponding conventional residual stresses) although much lower deformation rates are involved.

### 4.2. Realistic Thermomechanical Modeling of Extended Surface High-Coverage LSP Treatments with Explicit Consideration of the Viscous Drag Mechanism

This section presents the numerical–experimental results for realistic overlapped high-density treatments in Ti6Al4V. Both the thermal and mechanical effects are explicitly considered. Solid theoretical–simulation arguments support the idea of the discussed reduction in plastic strain. Nevertheless, proper experimental validation is required. Therefore, extended surface high-coverage LSP treatments are developed to achieve a double purpose: first, to provide a sufficiently large area to perform experimental measurements using the hole drilling method (experimental validation), and second, to provide an insight into the achievable results in realistic treatments when the viscous drag is explicitly considered. LSP is concerned with protecting extended surfaces susceptible to fatigue failure, and then concentric shots analysis is usually reserved for theoretical issues (Section 4.1). The very high-density treatment developed (the equivalent overlapping density (EOD) [48] is set as 5000 pp/cm^2^) is suitable to evidence the overestimated in-depth compressive residual stresses obtained using conventional modeling.

With the aid of a combined thermomechanical model, near-surface residual stress predictions are obtained (Section 4.2.1). Finally, the in-depth numerical–experimental results are presented in Section 4.2.2.

#### 4.2.1. Near-Surface Residual Stress Calculation

**Figure 6:**
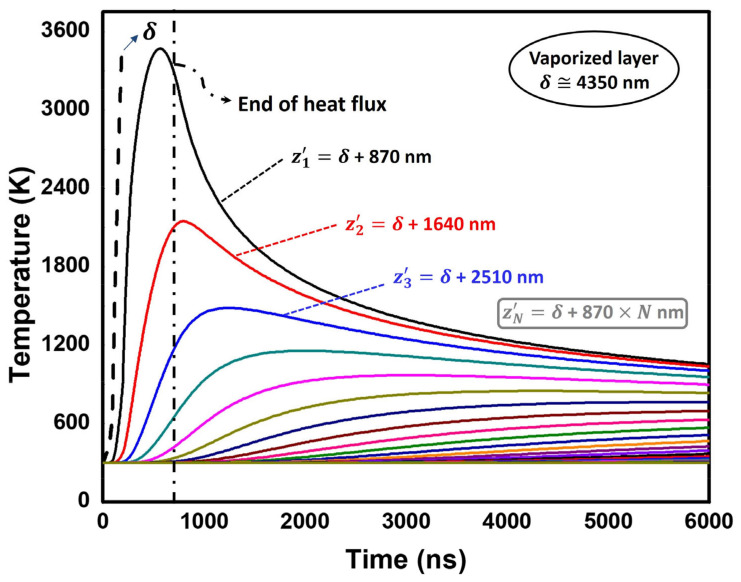
Time–temperature evolution for non-evaporated elements.

The heat flux (Table 1) is set as an input to calculate the temperature evolution for different depths (Figure 6). As expected, the results suggest that a thin layer may exceed the vaporization temperature. This result is consistent with the nature of LSP, in which evaporated material forms a plasma whose temperature increases up to 30,000 K. A simplified method is used to calculate material vaporization: if the surface element affected by irradiation reaches the vaporization temperature of the alloy (≅3500 K), the heat flux starts to affect the adjacent element. The results suggest that about the first δ ≅ 4.35 μm are vaporized per pulse, feeding the high-temperature plasma. It must be noted that the main purpose of the heat analysis is to obtain the time evolution of temperature near the surface, which is set as an input for plastic strain determination near the surface.

The in-depth residual stress predictions from 0 to 60 µm are one of the main issues to be discussed since cracks usually nucleate near the surface. The combination of mechanical + thermomechanical simulation provides an estimate of near-surface residual stresses (Table 3). The depths between $z_{1}^{\prime }$ and $z_{2}^{\prime }$ (Figure 6) are affected by temperatures between Ti6Al4V’s melting and vaporization points. Hence, the stress state after solidification along this thin layer may be particularly difficult to assess. Therefore, these depths have been neglected for the residual stress calculation. Very high tensile stresses are finally computed at the surface. This is motivated by a double effect caused by the local high temperatures involved: a temporal yield locus size reduction in combination with a notable thermal expansion. Hence, very high compressive strains are obtained near the surface, and consequently severe tensile stresses are computed. However, thermal effects are obtained within a very small depth (10 μm), beyond which high compressive residual stresses are predicted. Consequently, thermal effects are not expected to have a significant detrimental effect on fatigue life. Nevertheless, it must be noted that the thermal diffusivity in this particular alloy is relatively small, which leads to small thermally affected depths. On the other hand, thermal effects may have an increased impact, for instance, in aluminum alloys.

#### 4.2.2. In-Depth Numerical–Experimental Residual Stress Determination

**Figure 7:**
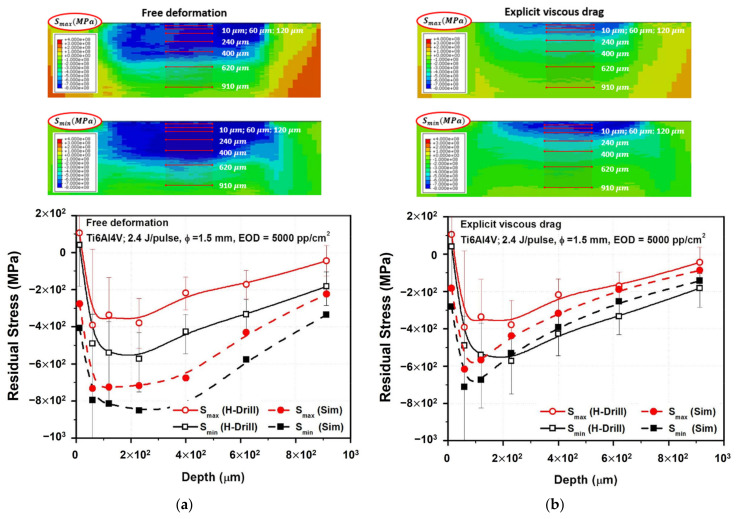
(**a**) Experimental vs. simulated in-depth residual stress profile predicted by conventional modeling. (**b**) Experimental vs. simulated in-depth residual stress profile with explicit consideration of viscous drag phenomenon.

The experimental validation of the above-presented results is hard to approach since the near-surface stress profile (0–10 μm) is beyond the hole drilling method’s accuracy. Therefore, to establish a consistent experimental–simulated comparison, the numerical results have been averaged inside each control volume removed by drilling. The near-surface simulated results are then softened using averaging inside the corresponding near-surface representative volume (first 20 μm). This makes possible a systematic experimental–simulated comparison by means of both models (Figure 7 and Table 4). The minimum in-plane stress, $S_{min}$, corresponds approximately to the stress along the peening direction, $S_{min}≅S_{PD}$. This is consistent with previous studies. Concerning the relative errors calculated in Table 4, it should be noted that high stress gradients are naturally presented near the surface. This effect may be enhanced by the low thermal diffusivity of Ti6Al4V alloy. In addition, surface detection is often hard to approach experimentally and, consequently, great uncertainties are calculated at the first microns. This leads to high numerical–experimental differences near the surface. Nevertheless, apart from quantitative differences, thermal simulation provides a qualitative explanation of the asymptote presented near the surface. 

Concerning the general trend of the in-depth residual stress profile, the results suggest that good agreement is obtained when the abrupt yield stress increase is considered (Figure 7b). On the other hand, overestimated general in-depth compressions are predicted by the conventional Johnson–Cook model (Figure 7a). Hence, the appropriateness of the explicit consideration of viscous drag stress in LSP simulations is experimentally confirmed. In summary, the hypothesis adopted (much lower plastic strain rates involved than the ones presented in conventional modeling) is consistent with the experiments. Although the computed strain rate is strongly reduced (10^4^ s^−1^ against conventionally reported 10^7^ s^−1^), the time window where plastic strain takes place is extended (during pressure decay, as plotted in Figure 3b), resulting in relatively precise in-depth compressive residual stress calculations, as demonstrated in the results plotted in Figure 7.

Concerning near-surface results in the range 0–60 μm, the numerical results show a strong gradient in the in-depth residual stress profile, in a consistent way with the experimental results, which may be softened in the experimental hole drilling measurements. This is not surprising since the hole drilling method is sensitive to near-surface strains and surface detection, leading to possible large uncertainties along the first microns. The X-ray diffraction method may be suitable for more precise results of the near-surface residual stress measurements.

## 5. Conclusions

In this paper, the relevance of the explicit consideration of the viscous drag deformation mechanism in the high-amplitude shockwaves generated by laser shock processing (LSP) has been demonstrated. The measured in-depth residual compressions in high-density treatments, obtained by means of the hole drilling method in Ti6Al4V alloy, confirm the accuracy of the proposed thermomechanical model and the appropriateness of precise yield stress modeling at high strain rates. The main conclusions are listed as follows:

a. The conventional models used in LSP modeling (i.e., the Johnson–Cook model) predict plastic strain rates of about 10^7^ s^−1^. However, the solid theoretical argument supports the idea of a notable reduction in the plastic strain rate to 10^4^ s^−1^ in alloys characterized by high yield stresses, for instance, Ti6Al4V alloy. The numerical–experimental results presented in this paper are consistent with this hypothesis.
b. The time pressure decrease plays an extremely important role in the deformation mechanism. In fact, the significant increase in the yield stress at the viscous regime (≅10^4^ s^−1^) imposes a threshold on the maximum plastic strain rate, and hence most of the deformation takes place during pressure decay, where two simultaneous conditions are presented: relatively high pressures with a smooth time evolution, which is responsible for extending the time during which deformation rates of about 10^4^ s^−1^ take place. This fact justifies that reasonable deformations are obtained (with their corresponding conventional residual stresses), although much lower deformation rates are involved. The numerical–experimental results confirm that notable overestimations in the in-depth compressions are predicted by means of conventional modeling in very high-density treatments, whereas good agreement is obtained when viscous drag is explicitly considered.
c. The calculated thermal effects in LSP induce significant reverse plastic straining through a very thin layer of material (about 10 μm is estimated for Ti6Al4V). This justifies the abrupt near-surface stress gradients: from very high tensile stresses (about the yield stress) at the surface to relatively high compressions (about −600 MPa) at 10 μm. Therefore, near-surface numerical–experimental validation can hardly be approached using the usual semi-destructive experimental residual stress measurements (i.e., the hole drilling method). X-ray diffraction methods may be suitable for this purpose.

## Figures and Tables

**Table 1:** Ti6Al4V sample composition.

Element	Al	V	C	O	N	Ti
Weight %	6.1	4.2	0.01	0.12	0.006	Bal.

**Table 2:** Time evolution of the irradiance, $I\left(t\right)$, heat flux, $q\left(t\right)/A$, and pressure, $P\left(t\right)$.

Time (ns)	Irradiation, $I\left(t\right)$ (GW/cm^2^)	Heat Flux, $q\left(t\right)/A$ (MW/cm^2^)	Pressure, $P\left(t\right)$ (MPa)
0	0	0	0
2	8	20	3370
5	20	50	5350
6	18	60	5183
10	11	110	4514
16	3	63	3511
23	0	42	2340
25	0	36	2177
30	0	32	1770
50	0	18	1160
100	0	9	840
300	0	3	617
400	0	2	506
770	0	0	0

**Table 3:** Simulated thermomechanical near-surface residual stresses.

Depth (nm)	$S_{min}$ (MPa)	$S_{max}$ (MPa)
0	1254	1398
870	849	1071
1740	473	657
2610	153	328
3480	−48	102
4350	−190	−44
5220	−308	−178
6090	−408	−296
6960	−504	−410
7830	−576	−499
8700	−609	−541
9570	−613	−544

**Table 4:** Numerical–experimental in-depth residual stress profiles.

Experimental Results			Free Deformation				Explicit Viscous Drag			
Depth (µm)	$S_{min\;exp}$ (MPa)	$S_{max\;exp}$ (MPa)	$S_{min\;sim}$ (MPa)	$S_{max\;sim}$ (MPa)	${err}_{min}$ %	${err}_{max}$ %	$S_{min\;sim}$ (MPa)	$S_{max\;sim}$ (MPa)	${err}_{min}$ %	${err}_{max}$ %
10	41	104	−405	−276	1088	365	−283	−181	790	274
60	−488	−392	−795	−732	63	87	−711	−619	46	58
120	−541	−336	−812	−723	50	115	−675	−566	25	68
240	−571	−379	−852	−717	49	89	−533	−438	7	16
400	−426	−218	−803	−678	88	211	−388	−316	9	45
620	−335	−176	−579	−434	73	147	−254	−188	24	7
910	−184	−48	−336	−227	83	373	−142	−86	23	79

## Data Availability

The data presented in this study are available on request from the corresponding author.
